# The negative pressure wound therapy for prevention of sternal wound infection: Can we reduce infection rate after the use of bilateral internal thoracic arteries? A systematic literature review and meta-analysis

**DOI:** 10.1186/s13019-024-02589-y

**Published:** 2024-02-12

**Authors:** Hind Elhassan, Ridha Amjad, Unna Palaniappan, Mahmoud Loubani, David Rose

**Affiliations:** 1 Cardiothoracic Department, Hull University Teaching Hospital NHS Foundation Trust, Castle Rd, Cottingham, East Riding of Yorkshire, HU16 5JQ UK; 2 Lancaster Medical School, Lancaster University, Lancaster, UK; 3 Cardiothoracic Department, Lancashire Cardiac Centre, Blackpool Teaching Hospital NHS Foundation Trust, Blackpool, UK

**Keywords:** Negative pressure wound therapy, Bilateral internal mammary artery, Sternal wound infections, Coronary artery bypass grafting

## Abstract

**Background:**

Negative pressure wound therapy (NPWT) is traditionally used to treat postoperative wound infections. However, its use in closed wound sternotomy post cardiac surgery in high-risk patients has become increasingly popular. The potential preventive benefit of reducing sternal wound infections has been recently acknowledged. Bilateral internal mammary artery (BIMA) grafts are used in coronary artery bypass grafting but have been associated with an increased risk of sternal wound infections (SWIs).

**Objectives:**

This systematic analysis examines whether NPWT can reduce the incidence of SWI following BIMA grafts, leading to more patients benefiting from the better survival outcome associated with BIMA grafting.

**Method:**

A comprehensive systematic search and meta-analysis were performed to identify studies on the use of NPWT in closed wound sternotomy. Ovid MEDLINE (in-process and other nonindexed citations and Ovid MEDLINE 1990 to present), Ovid EMBASE (1990 to present), and The Cochrane Library (Wiley), PubMed, and Google Scholar databases were searched from their inception to May 2022 using keywords and MeSH terms. Thirty-four articles from 1991 to May 2022 were selected.

**Result:**

Three studies reported on the outcome of NPWT following BIMA grafting. The pooled analysis did not show any significant difference in the incidence of sternal wound infection between NPWT and standard dressing (RR 0.48 95% CI 0.17–1.37; *P *= 0.17) with substantial heterogeneity (I^2^ 65%). Another seven studies were found comparing the outcome of SWI incidence of negative pressure closed wound therapy with conventional wound therapy in patients undergoing adult cardiac surgery. The pooled analysis showed that NPWT was associated with a low risk of SWIs compared to conventional dressing (RR 0.47 95% CI 0.36–0.59; *P *< 0.00001), with low heterogeneity (I^2^ 1%).

**Conclusion:**

The literature identified that NPWT significantly decreased the incidence of sternal wound complications when applied to sutured sternotomy incisions in high-risk patients, and in some cases, it eliminated the risk. However, the inadequate number of randomized controlled trials assessing the effectiveness of NPWT in BIMA grafting emphasizes the need for further, robust studies.

**Supplementary Information:**

The online version contains supplementary material available at 10.1186/s13019-024-02589-y.

## Introduction

Sternal wound infections (SWIs) can cause extensive complications following median sternotomy in cardiac operations. Studies have shown that the incidence of SWI after cardiac surgery ranges from 0.9% to 20%. 1.6% of these patients develop deep sternal wound infections (DSWIs), which can affect muscle tissue, sternum, sub sternum, and mediastinum [1–3]. According to Perezgrovas-Olaria et al. [1] on their recent meta-analysis deep sternal wound infection found to be associated with higher mortality, longer postoperative hospitalization, stroke, myocardial infarction and respiratory and renal failure. Superficial sternal wound infection, which involves the skin, subcutaneous tissue, and deep fascia [3]. Coronary artery bypass grafting (CABG) patients are susceptible to developing DSWIs and have been found to have a 2.5 times greater long-term mortality than those without any infection [4]. The literature has described numerous risk factors for SWIs [5]. The use of the bilateral internal mammary artery (BIMA) for coronary artery grafting is the most consistently reported independent risk factor for SWI, together with insulin-dependent diabetes and obesity BIMA use alone can increase the risk by 4.23 times [1, 5–10]. This has resulted in the use of BIMA in CABG, despite better long-term prognosis, which has been restricted to low-risk patients [1, 6, 11].

Traditionally, the use of NPWT has been restricted to treating wound complications with open post-sternotomy incisions [2, 11–13]. Further investigations into the use of NPWT have promoted its applicability as a prophylactic measure to reduce the incidence of SWI [2, 5]. Applying negative pressure to closed incisions promptly after surgery can prevent complications such as SWIs in high-risk individuals [2, 11].

**Fig. 1:**
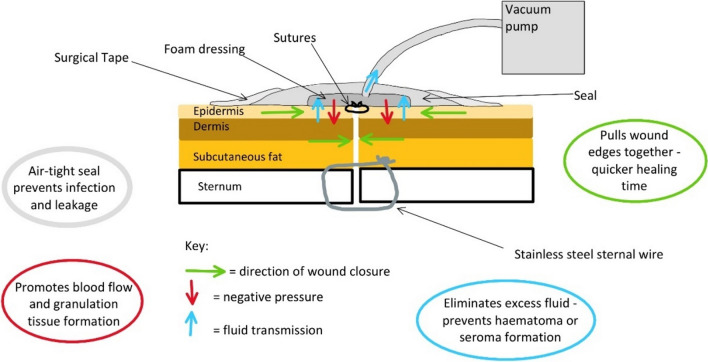
Illustration of mechanism through which NPWT prevent wound infection

NPWT arose in 1997 and has significantly benefited the outcomes of sternal wound dehiscence (SWD). The suction of excess tissue fluid facilitates the prevention of haematoma or seroma formation [14]. Negative pressure stimulates perfusion, resulting in an accelerated healing process in addition to a reduction in ischaemic wound necrosis, thereby preventing wound breakdown (Fig. 1) and helping in primary wound healing, especially in watershed regions [15].

If NPWT is proven effective, it can promote the practice of BIMA grafting, allowing long-term prognostic benefits [9, 10]. Therefore, this systematic literature review aims to examine the literature on the efficiency of NPWT in reducing the risk of SWI following BIMA grafts.

## Method

A systematic literature review according to the Preferred Reporting Items for Systematic Reviews and Meta-Analyses (PRISMA) was performed.

### Search strategy

A comprehensive systematic search was performed to identify studies that evaluated the usefulness of NPWT in preventing the risk of SWI post cardiac surgery. Ovid MEDLINE (in-process and other nonindexed citations and Ovid MEDLINE 1990 to present), Ovid EMBASE (1990 to present), and The Cochrane Library (Wiley), PubMed, and Google Scholar databases were searched from their inception to May 2022 using key words and the MeSH terms ‘cardiac surgery’, ‘negative pressure wound therapy’, ‘closed incision management’, ‘prevention’, ‘bilateral internal mammary arteries’, ‘BIMA’, and ‘sternal wound infection’.

### Study selection and inclusion criteria

Inclusion criteria were full text English studies where NPWT was used as prevention rather than as a treatment of postsurgical infections. Outcome measurements of sternal wound complications after application of the NPWT system in which BIMA had been used in coronary artery grafting. Studies that measure the outcome of NPWT in other cardiac surgeries have also been investigated. The reference lists were also searched for any relevant articles that met the criteria.

### Data extraction

Two authors independently extracted data (Elhassan H, Amjad R). Quality assessment was carried out by Elhassan (H) using the Newcastle‒Ottawa Scale (NOS) to assess the quality of nonrandomized studies in the meta-analysis (attached in supplementary material) [16].

### Statistical analysis

The primary endpoint was the incidence of sternal wound infection in NPWT compared to conventional therapy in patients who had bilateral mammary grafting. The secondary endpoint was the incidence of sternal wound infection in NPWT compared to conventional treatment in the general cardiac population. Categorical variables are expressed as percentages, and continuous variables are expressed as the mean and standard deviations. Random-effects models were used to consider pooled effects, heterogeneity and absolute values. The risk ratio (RR) with a 95% confidence interval (CI) was extracted from the incidence of SWIs. Data are summarized in tables and forest plots. Forest plots were used to determine the effect size. The I^2^ test was used to evaluate heterogeneity (unimportant (*I*^2^ = 0–40%), moderate (*I*^2^ = 40–60%), substantial (*I*^2^ = 60–75%), or considerable (*I*^2^ > 75–100%). Statistical analysis was performed using RevMan (Review Manager (RevMan) [Computer program]. Version 5.4. The Cochrane Collaboration, 2020).

## Results

**Fig. 2:**
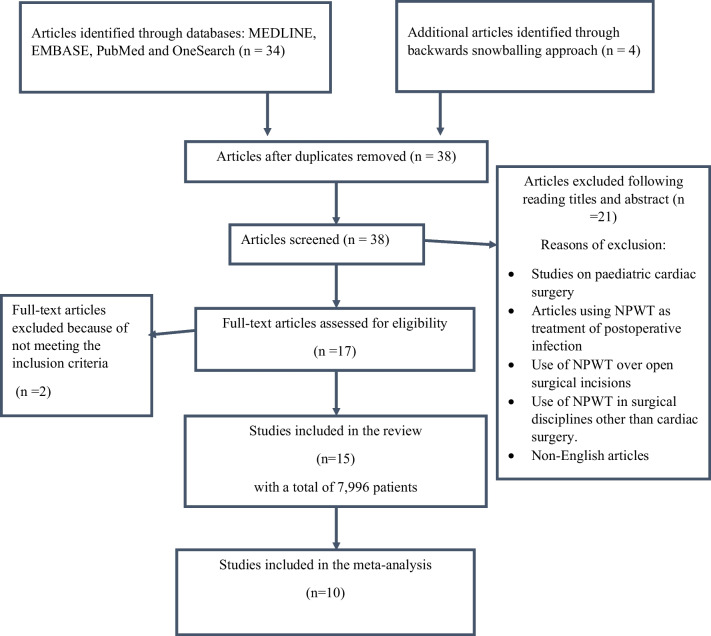
Flow diagram showing literature search methods for the systematic review

From 38 abstracts, 17 studies met the inclusion criteria for full-text review, out of which 15 studies were selected for systematic review and 10 studies for meta-analysis (Fig. 2).

Six out of the 15 studies addressed the outcome of NPWT in patients who underwent BIMA use for grafting. Nearly all the studies have reported favourable clinical outcomes apart from one that showed no difference in SWI incidence between NPWT and conventional therapy. We separated the studies’ results into two groups: studies addressing the outcome of NPWT following the use of BIMA for grafting and the remaining studies that investigated the outcome of NPWT in all other cardiac procedures without direct emphasis on BIMA in their analysis.

We identified a total of 7,996 patients, of whom 2,514 received NPWT post cardiac surgery on a closed incision; the included studies are summarized in Table 1.

**Table 1:** Characteristics of the studies included in the literature review and the meta-analysis

Study (Year of publication)	Country	Study design	Inclusion criteria	NPWT used	Standard dressing used	Duration of treatment (days)
Atkins et al. [5]	USA	Retrospective study	Diabetes, obesity, prolonged use of cardiopulmonary bypass and the need for intra-aortic balloon pump as well as the use of bilateral mammary arteries graft	VAC “(Kinetic Concepts Inc, San Antonio, TX)”	NA	2–4
Colli et al. [17]	Italy	Prospective study	Mean Fowler risk score 15.1 [8–30]	Prevena™	NA	5
Grauhan et al. [13]	Germany	Prospective study	BMI > 30 kg/m2, age 18 or more, absence of preoperative signs of inflammation	Prevena™	NA	6–7
Grauhan et al. [18]	Germany	Prospective study	NA	Prevena™	NA	6–7
Witt-Majchrzak et al. [19]	Poland	Prospective study	Off pump CABG	PICO™	NA	6
Santarpino et al. [20]	Germany	Retrospective study	Isolated CABG with BIMA grafting	Prevena™	Absorbent adhesive dressing (usually Cosmopor E (Paul Hartmann AG, Heidenheim, Germany)	5
Reddy [21]	USA	Retrospective study	At least 3 of the following: BMI > 35 kg/m2, diabetes, low albumin, poor tissue quality at incision, recent post operative infection, steroid, high dosage of pressor medication	Prevena™	NA	
Philip et al. [22]	Ireland	Retrospective study	obese, current smoker, hypertensive with COPD and diabetes mellitus	Prevena™	NA	6–7
Gatti et al. [7]	Italy	Prospective study	Isolated CABG with BIMA grafting	Prevena™	NA	NA
Ruggieri et al. [9]	France	Propensity match	Isolated CABG with BIMA grafting	Prevena™	Tegaderm (3 M Corporate, St. Paul, MN, USA)	5–7
Suleo-Calanao et al. [23]	United Kingdom	Retrospective study	NA	Prevena™	Opsite Postop (Smith + Nephew Inc., Andover, MA, USA)	5
Tabley et al. [24]	France	Retrospective study	NA	PICO™	NA	NA
Rashed et al. [25]	Hungary	Randomized trail	BMI > 30 kg/m2, diabetes, PVD, COPD	VivanoTec	NA	5
Brega et al. [26]	Italy	Prospective study	Two of the following criteria: diabetes on medical treatment, BMI > 30 kg/m2, COPD, diabetes, eGFR < 60 ml/min, BIMA grafting	AVELLE®	Hydrocolloid and carboxymethyl cellulose, or traditional gauzedressing	7
Nguyen et al. [27]	USA	Retrospective cohort study	All nontraumatic cardiothoracic surgery	Prevena™	NA	NA

Only 285 patients who had BIMA received NPWT, and the included studies are summarized in Tables 1 and 2.

**Table 2:** Study findings of the incidence of sternal wound infections after cardiac surgery with the use of NPWT

	No`. of patients (n)	Overall incidence No. (%)	No. of patients using standard treatment (n)	Specific incidence of SWI after standard treatment No. (%)	No. of patients using NPWT (n)	Specific incidence after NPWT No. (%)	P value
Atkins et al. [5]	57	0 (0%)	NA	NA	57	0 (0%)	NA
Colli et al. [17]	10	0 (0%)	N/A	N/A	10	0 (0%)	NA
Grauhan et al. [13]	150	15 (10%)	75	12 (16%)	75	3 (4%)	*P *= 0.0266
Grauhan et al. [18]	3745	122 (3.3%)	3508	119 (3.4%)	237	3 (1.3%)	*P *< 0.05
Witt-Majchrzak et al. [19]	80	13 (16.2%)	40	3 (7.5%)	40	10 (25%)	*P *= 0.03
Santarpino et al. [20]	129	6 (4.7%)	108	6 (5.6%)	21	0 (0%)	*P *= 0.336
Reddy [21]	27	4 (14.8%)	N/A	N/A	27	4 (14.8%)	NA
Philip et al. [22]	10	0 (0%)	NA	NA	10	0 (0%)	NA
Gatti et al. [7]	53	8 (15.1%)	NA	NA	53	8 (15.1%)	NA
Ruggieri et al. [9]	256	27 (10.5%)	128	14 (10.9%)	128	13 (10.2%)	*P *= 0.819
Suleo-Calanao et al. [23]	1859	122 (6.6%)	927	81 (8.7%)	932	41 (4.4%)	*P *= 0.0005
Tabley et al. [24]	233	25 (10.7%)	91	16 (17.6%)	142	9 (6.3%)	*P *= 0.009
Rashed et al. [25]	98	6 (6.3%)	52	6 (11.1%)	46	0 (0%)	*P *= 0.026
Brega et al. [26]	90	13	60	12	30	1	*P *= 0.06
Nguyen et al. [27]	1199	4.3%	493	31 (6.3%)	706	21 (3%)	*P *= 0.01

### Primary endpoint: outcome of NPWT following BIMA grafting

**Fig. 3:**
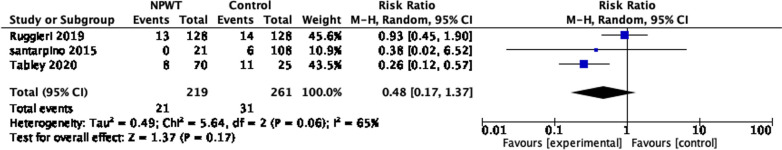
Sternal wound infection following bilateral mammary artery grafting

A total of six studies reported on the outcome of NPWT following BIMA grafting. Three studies investigated BIMA grafting as their primary endpoint, while the remaining studies had a subgroup analysis of patients with BIMA grafts from their study group. Three studies compared this outcome with the control group receiving conventional treatment. The quality of these studies was poor, and only one study used propensity-score matching. The pooled analysis did not show a significant difference between NPWT and standard dressing (RR 0.48 95% CI 0.17–1.37; *P *= 0.17), with substantial heterogeneity (I^2^ 65%) Fig. 3.

### Outcome of SWIs following NPWT in cardiac surgery

Seven further studies were found in the literature comparing the outcome of SWI incidence with negative pressure closed wound therapy with conventional wound therapy in patients undergoing adult cardiac surgery. We also examined these studies to explore the effect of NPWT in reducing the incidence of sternal wound infection in other high-risk cardiac patients.

**Fig. 4:**
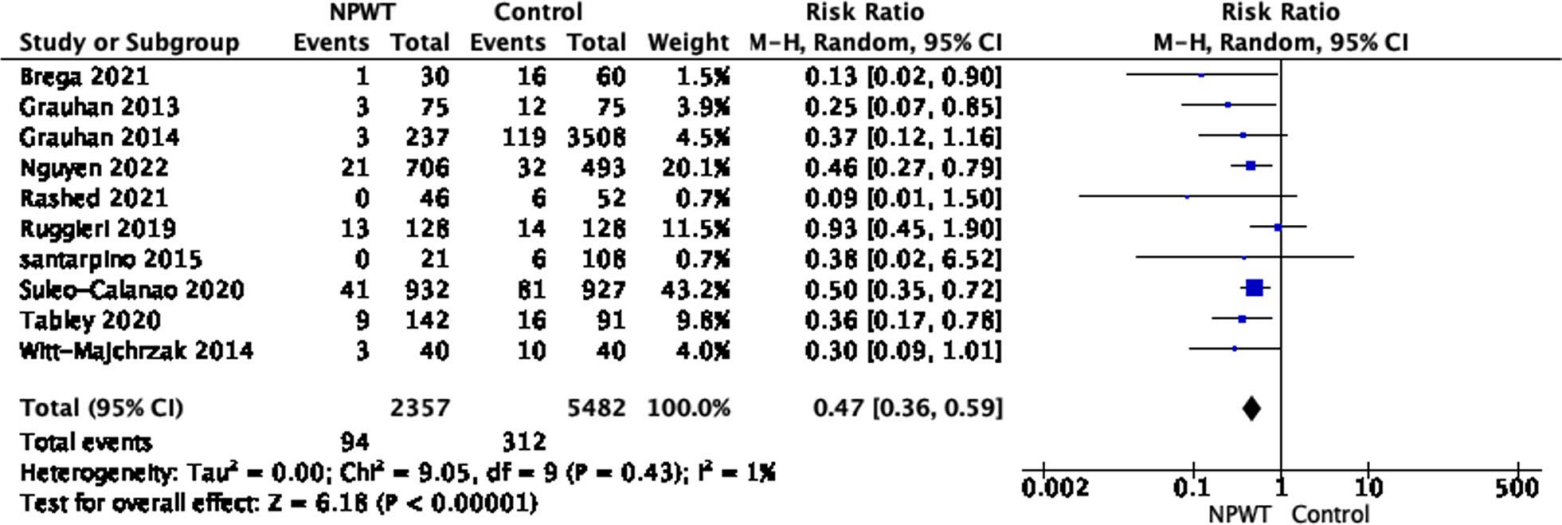
Sternal wound infection following general cardiac operations

Meta-analysis was conducted for all the studies with a comparison group (a total of 10 studies, including studies in the BIMA group). Two studies were randomized [19, 25], and one used propensity-score matching [9]. The pooled analysis showed that NPWT was associated with a low risk of SWIs compared to conventional dressing (RR 0.47 95% CI = 0.36–0.59; *P *< 0.00001), with low heterogeneity (I^2^ 6%) (Fig. 4).

**Fig. 5:**
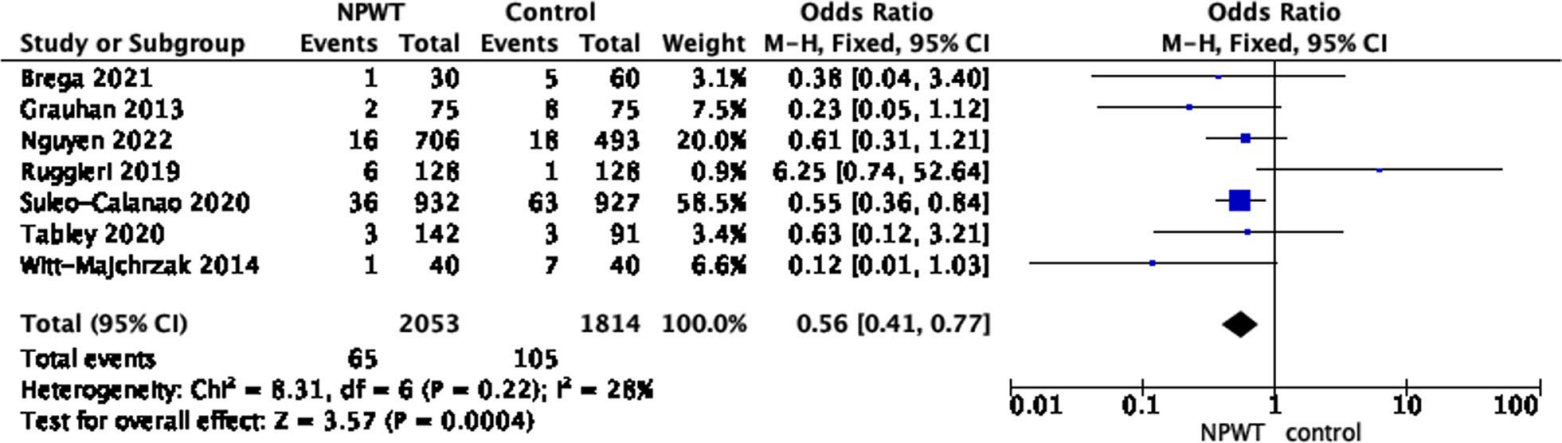
Superficial sternal wound infection (SSWI) post cardiac surgery

**Fig. 6:**
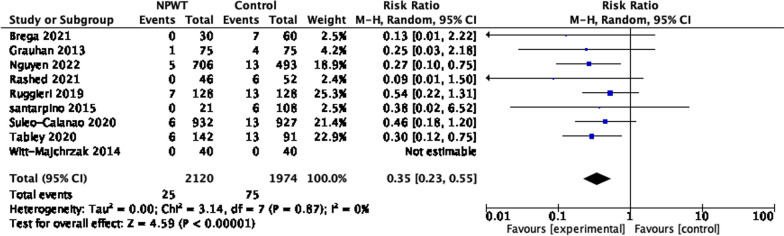
Deep sternal wound infection (DSWI) post cardiac surgery

We have run further subgroup analysis to investigate if NPWT have a preventive benefit on superficial and deep sternal wound infection (Figs. 5, 6).

Three studies were not included in the analysis of SSWI, as either did not report the number of SSWI separately in their studies [18, 20] or the study only focus on DSWI [25]. NPWT remains to have law risk of SSWI in comparsion with conventional dressing (RR 0.56 95% CI 0.41, 0.77; *P *= 0.0004) with unimportant heterogenicity I^2^ = 28%).

One study did not provide breakdown on number of patients with DSWI [18], and one study had no deep sternal wound infection in both arms of the study [19]. The RR 0.35 95% CI 0.23, 0.55; *P *< 0.00001 and no heterogenicity.

Fifteen studies met the inclusion criteria and were included in this review. Nine studies were included in the meta-analysis. The majority of the studies (12 articles) demonstrated a reduction in sternal wound complications in high-risk cardiac surgery associated with the administration of negative pressure on sutured incisions. Two studies reported no significant difference in the rate of SWI between patients receiving NPWT and those receiving conventional treatment. Six studies highlighted the outcome of NPWT within BIMA patients, whether it was part of a subgroup analysis or the primary treatment. Four studies reported positive patient outcomes associated with NPWT, while two articles with control patient groups showed no significant difference. However, pooled analysis was nonsignificant (RR 0.48 95% CI 0.17–1.37; *P *= 0.17), with substantial heterogeneity (I^2^ 65%). However, the general trend was the beneficial effect of NPWT across the different studies.

Atkins, Colli, Gatti, Reddy and Philip [5, 8, 21, 22] all demonstrated the potency of NPWT post-surgery, as patient reports were entirely clear of infection after prophylactic management. However, the absence of control groups and the small sample size makes it difficult to obtain significant results to draw a clear conclusion and limits the generalizability of these trials to the wider population. As these studies were not randomized controlled studies, it may be argued that the promising results discovered are not the sole by product of the negative pressure wound therapy system; other factors might be involved [11, 17]. The literature has shown that female sex increases the risk of postoperative complications [5]. Despite this, Atkins and colleagues failed to include sufficient females in their selected participants, reinforcing the reduced infection rate [5]. Thus, trials that consider the relative contributions of other risk factors in postoperative infection, with sufficient patients, are needed to fully understand the protective effect of negative pressure wound therapy on closed chest incisions.

## Discussion

BIMA use has been associated with increased graft patency and positive long-term clinical outcomes in many studies and literature review [28, 29]. Although this is the case, there has been some reluctance to use BIMA for coronary grafting, given the risk of sternal wound infection associated with its use. The Arterial Revascularisation Trial (ART), a multicentre, randomized controlled trial, did not show superiority of BIMA grafting compared to single internal mammary artery (SIMA) grafting with vein grafts after 10 years of follow-up [30]. However, this finding contradicted the largeevidence in the literature. Multiple factors may contribute to this finding: first, the study design was unblinded, surgeons’ experience was not taken into consideration, there was a lack of angiographic studies to assess graft patency, and there was nonadherence to randomization. The SIMA group received radial artery grafting as well, which is known to have a better outcome than vein graft [31]. Several studies reported higher survival rates and less reintervention compared to SIMA. Tatoulis et al. [29] conducted a large observational study evaluating the patency of the right internal mammary artery (RIMA) by performing coronary angiography for 991 patients out of 5,766 patients who underwent BIMA grafting in their institution. The ten-year overall patency was 90% for grafts to the left anterior descending artery (LAD) 95%, graft to the circumflex artery (Cx) 91%, graft to the right coronary artery (RCA) 84% and graft to the posterior descending artery (PDA) 86%. This was identical to the 10-year patency as LIMA to LAD and LIMA to Cx. They also demonstrate better RIMA patency than radial artery and vein grafts. There was an 89% survival rate for patients who had BIMA grafting. A meta-analysis of 8 propensity-matched studies by Gaudino et al. [28] showed that BIMA grafting is associated with superior long-term survival irrespective of sex and diabetes. Davierwala et al. [32] recently published their outcome of using BIMA in minimally invasive off-pump coronary artery grafts; early outcomes showed 96.8% graft patency on angiographic studies prior to discharge.

Research evidence has shown that using IMA grafts (or ITA grafts) correlates with an elevated risk of SWI, especially when using BIMA [6]. Research by Grossi et al. [33] demonstrated that having one or more IMA grafts increased the prevalence of sternal infection by 1.37% compared to patients who had no IMA graft in CABG surgery. A BIMA graft was associated with a 1.7% higher prevalence of SWI than a single IMA graft (SIMA), and the difference in prevalence increased to 8.8% when combined with diabetes. TheART-reported incidence of sternal wound complications was almost double in the BIMA group than in the SIMA group (54 cases vs. 30 cases) after a 10-year follow-up, and there was a significantly increased rate of sternal reconstruction in the BIMA group (31 cases vs. 10 cases) [30]. Ståhle et al. [34] investigated sternal wound problems after cardiac surgery in a large cohort of 13,285 patients over fifteen years. Of these, 155 patients had BIMA grafting, with a sternal wound infection rate of 3.9%; this was more than 2 times the rate in other cases (1.7%) [34]. The results from a prospective study using patients from ten surgical institutions in Paris (n = 1830) showed that the incidence of DSWI in BIMA grafting patients was 6% higher than that in SIMA patients (8.7% vs. 2.7%) [35]. The robust association between BIMA use and the incidence of SWIs has been determined by large randomized clinical trials, with infection rates ranging from 2.1 to 8.7% [8, 10, 33–37]. As a result, it is critical to acknowledge other risk factors (e.g., obesity, diabetes) when using bilateral internal mammary arteries so that strategies to adjust for these risk factors can be implemented in vulnerable patients before surgery [38]. The use of negative pressure wound therapy might be particularly beneficial in this patient category.

The NPWT system uses special dressings and a negative pressure-creating device to provide a proportional distribution of negative pressure over closed surgical sites [2, 5, 11]. The wound site and surrounding skin are coveredwith an adherent sterile cover, and a vacuum pump is joined to the dressing by a suction tube [11]. This gives negative pressures ranging from − 75 to − 125 mmHg, enabling wound fluid to be drained into an aseptic canister, as in the case of Prevena™ dressing [2, 5, 7, 11, 17]. There are several mechanisms through which NPWT devices exert their effects. It has been suggested that introducing a closed wound medium keeps the sealed incision margins composed, stimulates cells to proliferate and triggers angiogenesis [2, 5, 7, 11, 13, 39]. The extracellular fluid is drained, allowing the removal of exudative content and tissue oedema. Blood flow to the wound area is increased, thus enhancing tissue perfusion and improving the circulation of immune cells and antibiotics [2, 7, 11, 13, 39]. These factors prevent the progression of infection and the incidence of sternal wound complications by deterring colony-forming bacteria and increasing the production of granulation tissue [2, 7, 11, 13, 39]. There have been few NPWT systems on the market, with Prevena™ currently being the most researched system [7, 9, 13, 17–22, 27]. Witt-Majchrzak et al. [19] reported in their study the NPWT has reduced the risk of overall sternal wound complications including sternal wound instabilities and abnormal healing.

In this meta-analysis, NPWT was shown to have a preventive benefit for wound infection post BIMA use in patients who underwent coronary artery bypass graft and in all other adult cardiac surgeries.

The pooled analysis for studies in general adult cardiac surgery showed a significant reduction in wound infection in patients who received negative pressure wound therapy compared to those who received conventional wound dressing (RR 0.47 95% CI 0.36–0.59; *P *< 0.00001), with low heterogeneity (I^2^ 6).

Negative pressure-generating interventions usually involve greater costs than conventional treatment [9]. Research by Tabley et al. showed that the total cost of buying PICO™ systems and the reduced costs of treating complications approximately saved £1188.79 “per patient” [24, 40]. Concerning the broader employment of NPWT in cardiac surgery, it is crucial to consider the economic effects of preventing SWIs versus treating the added complications [9, 23]. If additional analyses of the saved expenses are produced in the literature, they can further support the worthiness of NPWT in surgery.

## Study limitations

The majority of the studies were observational studies, apart from one randomized control trial and one propensity-score matching analysis to account for selection bias. The lack of robust studies affects the quality of the review due to literature biases. The small number of studies (n = 3) focusing specifically on the efficacy of prophylactic NPWT after BIMA grafting included in the final analyses (with high heterogeneity) makes it difficult to conclude the suitability of NPWT. We did not acknowledge the presence of treatment allocation bias. The use of NPWT is usually reserved for patients who are perceived to have a high surgical risk of developing SWI. Nonetheless, pooled analysis from studies covering all cardiac procedures showed a potential benefit of NPWT in preventing SWI. Finally, there was a language bias, as we only included studies in English.

## Conclusion

Based on findings from this systematic literature review and meta-analysis, closed incision management after cardiac surgery can reduce the incidence of sternal wound infections in cardiac patients. However, the potential usefulness of NPWT after bilateral internal mammary artery grafting needs to be further investigated. The inconsistent study findings in these patients and the flawed study designs leave the question regarding the clinical efficacy of prophylactic use of closed incisional negative pressure therapy in BIMA grafting not conclusively answered. Large-scale, randomized controlled studies that specifically measure postsurgical outcomes in BIMA patients following negative pressure wound therapy are needed.

### Supplementary Information

**Additional file 1. **Newcastle‒Ottawa Scale Qauality Assessment for included articles.

## Data Availability

The datasets supporting the conclusions of this article are included within the article (and its Additional file 1).

## Abbreviations

ART: Arterial revascularization trial
BIMA: Bilateral internal mammary artery
CABG: Coronary artery bypass grafting
Cx: Circumflex artery
DSWI: Deep Sternal Wound Infection
IMA: Internal mammary artery
ITA: Internal thoracic artery
LAD: Left anterior descending artery
LIMA: Left internal mammary artery
NOS: Newcastle‒Ottawa Scale
NPWT: Negative Pressure Wound Therapy
PDA: Posterior descending artery
RCA: Right coronary artery
RIMA: Right internal mammary artery
SSWI: Superficial sternal wound infection
SWIs: Sternal Wound Infections
SWD: Sternal wound dehiscence
PRISMA: Systematic Reviews and Meta-Analyses
SIMA: Single internal mammary artery

## References

[1] Perezgrovas-Olaria R, Audisio K, Cancelli G, Rahouma M, Ibrahim M, Soletti GJ, Chadow D, Demetres M, Girardi LN, Gaudino M (2023). Deep sternal wound infection and mortality in cardiac surgery: a meta-analysis. Ann Thorac Surg.

[2] Dohmen PM, Markou T, Ingemansson R, Rotering H, Hartman JM, van Valen R (2014). Use of incisional negative pressure wound therapy on closed median sternal incisions after cardiothoracic surgery: clinical evidence and consensus recommendations. Med Sci Monit.

[3] Song Y, Chu W, Sun J (2023). Review on risk factors, classification, and treatment of sternal wound infection. J Cardiothorac Surg.

[4] Toumpoulis IK, Anagnostopoulos CE, DeRose JJ, Swistel DG (2005). The impact of deep sternal wound infection on long-term survival after coronary artery bypass grafting. Chest.

[5] Atkins BZ, Wooten MK, Kistler J, Hurley K, Hughes CG, Wolfe WG (2009). Does negative pressure wound therapy have a role in preventing poststernotomy wound complications?. Surg Innov.

[6] Bayer N, Hart WM, Arulampalam T, Hamilton C, Schmoeckel M (2020). Is the use of BIMA in CABG sub-optimal? A review of the current clinical and economic evidence including innovative approaches to the management of mediastinitis. Ann Thorac Cardiovasc Surg.

[7] Gatti G, Ledwon M, Gazdag L, Cuomo F, Pappalardo A, Fischlein T (2018). Management of closed sternal incision after bilateral internal thoracic artery grafting with a single-use negative pressure system. Updates Surg.

[8] Gatti G, Benussi B, Brunetti D, Ceschia A, Porcari A, Biondi F (2018). The fate of patients having deep sternal infection after bilateral internal thoracic artery grafting in the negative pressure wound therapy era. Int J Cardiol.

[9] Ruggieri VG, Olivier ME, Aludaat C, Rosato S, Marticho P, Saade YA (2019). Negative pressure versus conventional sternal wound dressing in coronary surgery using bilateral internal mammary artery grafts. Heart Surg Forum..

[10] Borger MA, Rao V, Weisel RD, Ivanov J, Cohen G, Scully HE (1998). Deep sternal wound infection: risk factors and outcomes. Ann Thorac Surg.

[11] Scalise A, Calamita R, Tartaglione C, Pierangeli M, Bolletta E, Gioacchini M (2016). Improving wound healing and preventing surgical site complications of closed surgical incisions: a possible role of Incisional Negative Pressure Wound Therapy. A systematic review of the literature. Int Wound J..

[12] Huang C, Leavitt T, Bayer LR, Orgill DP (2014). Effect of negative pressure wound therapy on wound healing. Curr Probl Surg..

[13] Grauhan O, Navasardyan A, Hofmann M, Müller P, Stein J, Hetzer R (2013). Prevention of poststernotomy wound infections in obese patients by negative pressure wound therapy. J Thorac Cardiovasc Surg.

[14] Dohmen PM, Misfeld M, Borger MA, Mohr FW (2014). Closed incision management with negative pressure wound therapy. Expert Rev Med Devices.

[15] Atkins BZ, Tetterton JK, Petersen RP, Hurley K, Wolfe WG (2011). Laser Doppler flowmetry assessment of peristernal perfusion after cardiac surgery: beneficial effect of negative pressure therapy. Int Wound J.

[16] Ottawa Hospital Research Institute. [cited 2022 Oct 24]. Available from: https://www.ohri.ca/programs/clinical_epidemiology/oxford.asp

[17] Colli A (2011). First experience with a new negative pressure incision management system on surgical incisions after cardiac surgery in high risk patients. J Cardiothorac Surg..

[18] Grauhan O, Navasardyan A, Tutkun B, Hennig F, Müller P, Hummel M (2014). Effect of surgical incision management on wound infections in a poststernotomy patient population. Int Wound J.

[19] Witt-Majchrzak A, Zelazny P, Snarska J (2015). Preliminary outcome of treatment of postoperative primarily closed sternotomy wounds treated using negative pressure wound therapy. Pol Prz Chir Polish J Surg.

[20] A Retrospective Study to Evaluate Use of Negative Pressure Wound Therapy in Patients Undergoing Bilateral Internal Thoracic Artery Grafting. 27763880

[21] Reddy VS (2016). Use of closed incision management with negative pressure therapy for complex cardiac patients. Cureus..

[22] Philip B, McCluskey P, Hinchion J (2017). Experience using closed incision negative pressure wound therapy in sternotomy patients. J Wound Care.

[23] Suelo-Calanao RL, Thomson R, Read M, Matheson E, Isaac E, Chaudhry M (2020). The impact of closed incision negative pressure therapy on prevention of median sternotomy infection for high risk cases: A single centre retrospective study. J Cardiothorac Surg.

[24] Tabley A, Aludaat C, le Guillou V, Gay A, Nafeh-Bizet C, Scherrer V (2020). A survey of cardiac surgery infections with PICO negative pressure therapy in high-risk patients. Ann Thorac Surg..

[25] Rashed A, Csiszar M, Beledi A, Gombocz K (2021). The impact of incisional negative pressure wound therapy on the wound healing process after midline sternotomy. Int Wound J.

[26] Brega C, Calvi S, Albertini A (2021). Use of a negative pressure wound therapy system over closed incisions option in preventing poststernotomy wound complications. Wound Repair Regener.

[27] Nguyen KA, Taylor GA, Webster TK, Jenkins RA, Houston NS, Kahler DL (2022). Incisional negative pressure wound therapy is protective against postoperative cardiothoracic wound infection. Ann Plast Surg.

[28] Gaudino M, Puskas JD, di Franco A, Ohmes LB, Iannaccone M, Barbero U (2017). Three arterial grafts improve late survival: a meta-analysis of propensity-matched studies. Circulation.

[29] Tatoulis J, Buxton BF, Fuller JA (2011). The right internal thoracic artery: the forgotten conduit5,766 patients and 991 angiograms. Ann Thorac Surg.

[30] Taggart DP, Benedetto U, Gerry S, Altman DG, Gray AM, Lees B (2019). Bilateral versus single internal-thoracic-artery grafts at 10 years. N Engl J Med.

[31] Gaudino M, Benedetto U, Fremes S, Biondi-Zoccai G, Sedrakyan A, Puskas JD (2018). Radial-artery or saphenous-vein grafts in coronary-artery bypass surgery. N Engl J Med.

[32] Davierwala PM, Verevkin A, Sgouropoulou S, Hasheminejad E, von Aspern K, Misfeld M (2021). Minimally invasive coronary bypass surgery with bilateral internal thoracic arteries: early outcomes and angiographic patency. J Thorac Cardiovasc Surg.

[33] Grossi EA, Esposito R, Harris LJ, Crooke GA, Galloway AC, Colvin SB (1991). Sternal wound infections and use of internal mammary artery grafts. J Thorac Cardiovasc Surg.

[34] Ståhle E, Tammelin A, Bergström R, Hambreus A, Nyström SO, Hansson HE (1997). Sternal wound complications–incidence, microbiology and risk factors. Eur J Cardiothorac Surg.

[35] Taggart DP, Altman DG, Gray AM, Lees B, Gerry S, Benedetto U (2016). Randomized trial of bilateral versus single internal-thoracic-artery grafts. N Engl J Med.

[36] Gatti G, Dell’Angela L, Barbati G, Benussi B, Forti G, Gabrielli M (2016). A predictive scoring system for deep sternal wound infection after bilateral internal thoracic artery grafting. Eur J Cardiothorac Surg.

[37] Lemaignen A, Birgand G, Ghodhbane W, Alkhoder S, Lolom I, Belorgey S (2015). Sternal wound infection after cardiac surgery: incidence and risk factors according to clinical presentation. Clin Microbiol Infect.

[38] Lu JCY, Grayson AD, Jha P, Srinivasan AK, Fabri BM (2003). Risk factors for sternal wound infection and mid-term survival following coronary artery bypass surgery. Eur J Cardiothorac Surg.

[39] Strugala V, Martin R (2017). Meta-analysis of comparative trials evaluating a prophylactic single-use negative pressure wound therapy system for the prevention of surgical site complications. Surg Infect (Larchmt).

[40] New study demonstrates PICO◊ Single Use Negative Pressure Wound Therapy System (sNPWT) beats standard dressings in reducing surgical site complications resulting in fewer deep sternal wound infections (DSWI) following cardiac surgery | Smith & Nephew. Available from: https://www.smith-nephew.com/news-and-media/media-releases/news/new-study-demonstrates-pico-single-use-negative-pressure-wound-therapy-system-snpwt-beats-standard-dressings-in-reducing-surgical-site-complications-resulting-in-fewer-deep-sternal-wound-infections-dswi-following-cardiac-surgery

