# Supplementary material for: The negative pressure wound therapy for prevention of sternal wound infection: Can we reduce infection rate after the use of bilateral internal thoracic arteries? A systematic literature review and meta-analysis
Source: J Cardiothorac Surg. 2024 Feb 12;19:87. doi: 10.1186/s13019-024-02589-y (PMC10860270; doi:10.1186/s13019-024-02589-y)
Supplement: Supplementary file 1 — Additional file 1. Newcastle‒Ottawa Scale Qauality Assessment for included articles. [file 13019_2024_2589_MOESM1_ESM.docx]

**Supplementary material**

Supplementary Table 1. Newcastle-Ottawa Scale for studies included in the meta-analysis

| AUTHOR | SCORE | SELECTION | COMPARABILITY | OUTCOME/EXPOSURE |
| --- | --- | --- | --- | --- |
| Grauhan *et al* (2013) | 9 | **** | ** | *** |
| Grauhan *et al* (2014) | 6 | ** | ** | ** |
| Santaprino *et al* (2015) | 7 | **** | ** | * |
| Ruggieri *et al* (2019) | 7 | **** | ** | * |
| Suleo-Calanao *et al* (2020) | 6 | *** | ** | * |
| Tabley *et al* (2020) | 6 | *** | ** | * |
| Rashed *et al* (2021) | 8 | **** | ** | ** |
| Brega *et al* (2021) | 9 | **** | ** | *** |
| Nguyen *et al* (2022) | 7 | **** | ** | ** |
